# Prevalence and relationship of endosymbiotic *Wolbachia *in the butterfly genus *Erebia*

**DOI:** 10.1186/s12862-021-01822-9

**Published:** 2021-05-21

**Authors:** Kay Lucek, Selim Bouaouina, Amanda Jospin, Andrea Grill, Jurriaan M. de Vos

**Affiliations:** 1 Department of Environmental Sciences - Botany, University of Basel, Schönbeinstrasse 6, CH- 4056 Basel, Switzerland; 2 Laboratory of Functional Ecology, Institute of Biology, University of Neuchâtel, Rue Emile-Argand 11, 2000 Neuchâtel, Switzerland; 3 Institute of Ecology and Evolution, University of Bern, Baltzerstrasse 6, CH-3012 Bern, Switzerland

**Keywords:** Lepidoptera, *Wolbachia*, Host shift, Endosymbiont, Coevolution, Glacial refugia

## Abstract

**Background:**

*Wolbachia* is an endosymbiont common to most invertebrates, which can have significant evolutionary implications for its host species by acting as a barrier to gene flow. Despite the importance of *Wolbachia*, still little is known about its prevalence and diversification pattern among closely related host species. *Wolbachia* strains may phylogenetically coevolve with their hosts, unless horizontal host-switches are particularly common. We address  these issues in the genus *Erebia,* one of the most diverse Palearctic butterfly genera.

**Results:**

We sequenced the *Wolbachia* genome from a strain infecting *Erebia cassioides* and showed that it belongs to the *Wolbachia* supergroup B, capable of infecting arthropods from different taxonomic orders. The prevalence of *Wolbachia* across 13 closely related *Erebia* host species based on extensive population-level genetic data revealed that multiple *Wolbachia* strains jointly infect all investigated taxa, but with varying prevalence. Finally, the phylogenetic relationships of *Wolbachia* strains are in some cases significantly associated to that of their hosts, especially among the most closely related *Erebia* species, demonstrating mixed evidence for phylogenetic coevolution.

**Conclusions:**

Closely related host species can be infected by closely related *Wolbachia* strains, evidencing some phylogenetic coevolution, but the actual pattern of infection more often reflects historical or contemporary geographic proximity among host species. Multiple processes, including survival in distinct glacial refugia, recent host shifts in sympatry, and a loss of *Wolbachia* during postglacial range expansion seem to have jointly shaped the complex interactions between *Wolbachia* evolution and the diversification of its host among our studied *Erebia* species.

**Supplementary Information:**

The online version contains supplementary material available at 10.1186/s12862-021-01822-9.

## Background

*Wolbachia* is one of the most common endosymbiotic bacteria that terrestrial arthropods harbor, estimated to occur in about half of all terrestrial arthropod species [1]. *Wolbachia* can affect their hosts in diverse ways*,* and may even trigger host diversification and speciation [2, 3]. *Wolbachia* can for example lead to sex-ratio distortions through male-killing [4, 5] or result in cytoplasmic incompatibility, whereby a female can only mate with uninfected males or males that carry a matching *Wolbachia* strain, contributing to the spread of *Wolbachia* [6, 7]. In the latter case, *Wolbachia* infection can cause reproductive isolation or intraspecific differentiation by reducing gene flow between different host populations. The impact of *Wolbachia* varies however among host species and is in most cases not known. The prevalence of *Wolbachia* may similarly differ among species, across a species range or seasonally within a species [1, 8, 9]. The turnover of *Wolbachia* has been suggested to be high, because closely related host species can harbor very divergent *Wolbachia* strains and due to differing *Wolbachia* prevalence [3, 10].

Infection by *Wolbachia* is estimated to be very abundant in Lepidoptera, with more than 160,000 described species of butterflies and moths, one of the most diverse groups of arthropods [11]. It may occur in up to 80% of all studied Lepidoptera taxa but with significant variation among the taxonomic families investigated so far [10, 12]. As for other groups of arthropods, *Wolbachia* in Lepidoptera has been shown to for example result in feminization [13], male-killing [14] or cytoplasmic incompatibilities [15]. However, to which degree *Wolbachia* may have contributed to the spectacular diversification of Lepidoptera remains elusive. Divergent *Wolbachia* infections may for example act as a barrier to gene flow upon secondary contact between closely related host lineages or species [16], as has been suggested in some cases [17, 18]. A first step to assess the potential evolutionary implications of *Wolbachia* is the quantification of its prevalence and differentiation among closely related host species. This would reveal how the divergence among hosts, or alternatively, their geographic proximity, affects the probability of sharing the same *Wolbachia* strains [3]. Here, we study the prevalence, diversity and relationships among *Wolbachia* strains in one of the most species-rich Palearctic butterfly genera—*Erebia*.

*Erebia* is a genus of cold-adapted butterflies for which mountainous environments represent diversity hotspots [19, 20]. The diversification of *Erebia* has been associated with differentiation in distinct glacial refugia as a consequence of the Quaternary glacial cycles [21–23]. While distantly related species often coexist and exploit different microhabitats following their postglacial range expansions [19, 24], in several cases closely related species or lineages exclude each other by forming very narrow secondary contact zones [18, 19]. Despite their broad Palearctic distribution, relatively little is known about the diversity and actual impact of *Wolbachia* in this host genus. In the case of *E. tyndarus* and *E. cassioides*, the secondary contact zone manifests as a very narrow genomic cline in the central Alps with only few F1 hybrid individuals, suggesting selection against interspecific gene flow in this system [18]. *Wolbachia* might represent an important barrier separating the two species, given that the genomic cline overlaps with changes in *Wolbachia* prevalence: While more than 90% of *E. cassioides*, including the putative F1 hybrids, were infected*,* none of the studied *E. tyndarus* specimens carried *Wolbachia* in the contact zone [18]. Divergent *Wolbachia* infections could therefore be one of the factors that underlie diversification of *Erebia.* *Erebia* species may also be infected by different *Wolbachia* strains simultaneously, as has been found for *E. aethiops* in Siberia [10].

To further advance our understanding on whether and how *Wolbachia* may drive the diversity of Lepidopteran clades, our aim was to characterize the prevalence and diversity of *Wolbachia* infecting *Erebia* species that often coexist in close proximity*.* Using long-read sequencing, we first assembled and annotated a reference genome for *Wolbachia* from the host *Erebia cassioides.* We then phylogenetically placed this strain among other, already sequenced *Wolbachia* strains that infect arthropods taking advantage of already existing annotated *Wolbachia* reference genomes as well as a larger Multi-Locus Sequence Typing (MLST) dataset*.* Next, we analyzed extensive population genomic data for 13 *Erebia* species and lineages, representing a small subset of the more than a hundred described *Erebia* species [20, 25], and assessed the prevalence of *Wolbachia* among different host species. Finally, we tested for phylogenetic coevolution of *Erebia* hosts and their *Wolbachia* strains, and placed putative host shifts in a geographic context*.* Overall, our results highlight that various factors shape the cryptic diversity of *Wolbachia* in *Erebia.*

## Results

### The Wolbachia genome

Our assembled *Wolbachia* genome was 1,423,447 bps long and consisted of two contigs of 1,166,489 and 256,958 bps length, respectively, and a GC content of 34.1% (Additional file 2: Table S1). The Rapid Annotation using Subsystem Technology (RAST) annotated features include 1436 putative protein-coding genes with 36 RNAs elements. A total of 326 genes (23%) could be assigned to a subsystem, i.e. proteins grouped by a relationship in function (Additional file 1: Figure S1). The 1436 predicted protein coding genes in our *Wolbachia* genome contained 172 complete and single copy BUSCO groups, no complete and duplicated BUSCOs, 5 fragmented BUSCOs, and 44 missing BUSCOs, resulting in a 77.8% BUSCO completeness score, which is comparable to other *Wolbachia* assemblies [26].

**Fig. 1:**
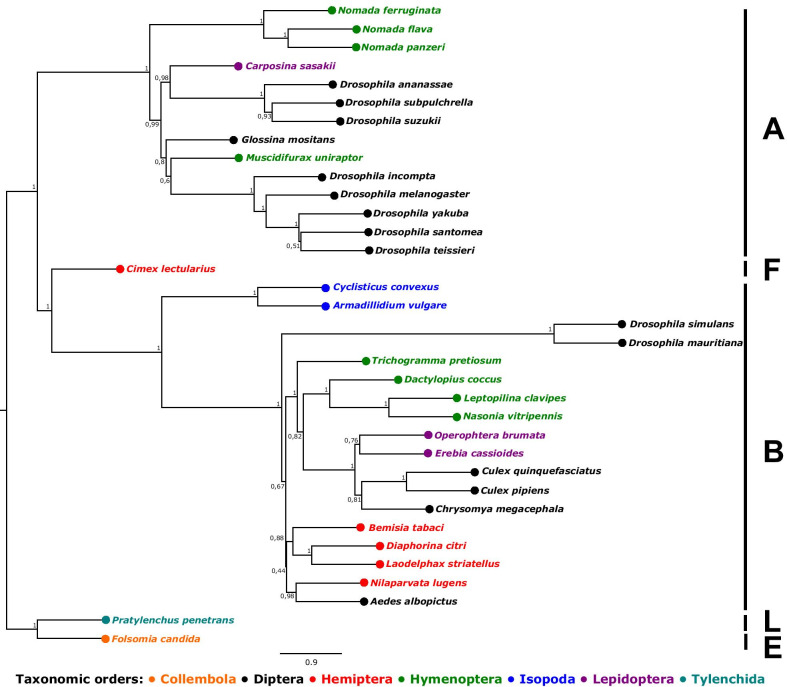
Astral summary phylogeny based on maximum likelihood gene trees from 184 single copy orthologs obtained from 36 *Wolbachia* genomes, including the newly sequenced strain from *Erebia cassioides*. Labels indicate the host species of each *Wolbachia* strain (see Table S1). The phylogeny was arbitrarily rooted on the branch separating Collembola and Tylenchida (Nematoda) from all other arthropods. Colors depict taxonomic orders and numbers at nodes indicate branch support in local posterior probability. Branch length in coalescence units, with terminal branches arbitrarily set at the default value 10. Capital letters depict *Wolbachia* supergroups (taken from [56])

A total of 184 single copy orthologs were identified across the 36 *Wolbachia* strains that we included in our study. Their coalescent-based species tree was generally well supported and clustered by the different *Wolbachia* supergroups, which corresponded though poorly to taxonomic orders of the host species (Fig. 1). The newly sequenced *Wolbachia* strain from *E. cassioides* clustered within the common supergroup B, next to the strain of the winter moth *Operophtera brumata*, which shows similarities to the *wPip Wolbachia* strain found in the common house mosquito *Culex pipiens* [27].

The sequences of all MLST loci that we extracted from our *Wolbachia* genome differed from publicly available sequences (Additional file 1: Figure S2–S6). Maximum likelihood trees of all five MLST loci, each including all available alleles recovered from arthropod hosts plus our newly sequenced genome, revealed that our assembled *Wolbachia* genome was deeply nested within *Wolbachia* supergroup B, confirming the relations among the single copy orthologs (Fig. 1). The relations among accessions of MLST loci also corresponded poorly to the taxonomic orders of the host species, indicative of pervasive host shifts. The closest related *Wolbachia* allele was found in spider mites (Trombidiformes, Acari) for the gene *coxA*; in spider mites and other arthropods (genes *hcpA* and *fbpA*); in Lepidoptera (*gatB*); or in Lepidoptera and other arthropods (*ftsZ*).

### *Wolbachia* abundance and coevolution

**Fig. 2:**
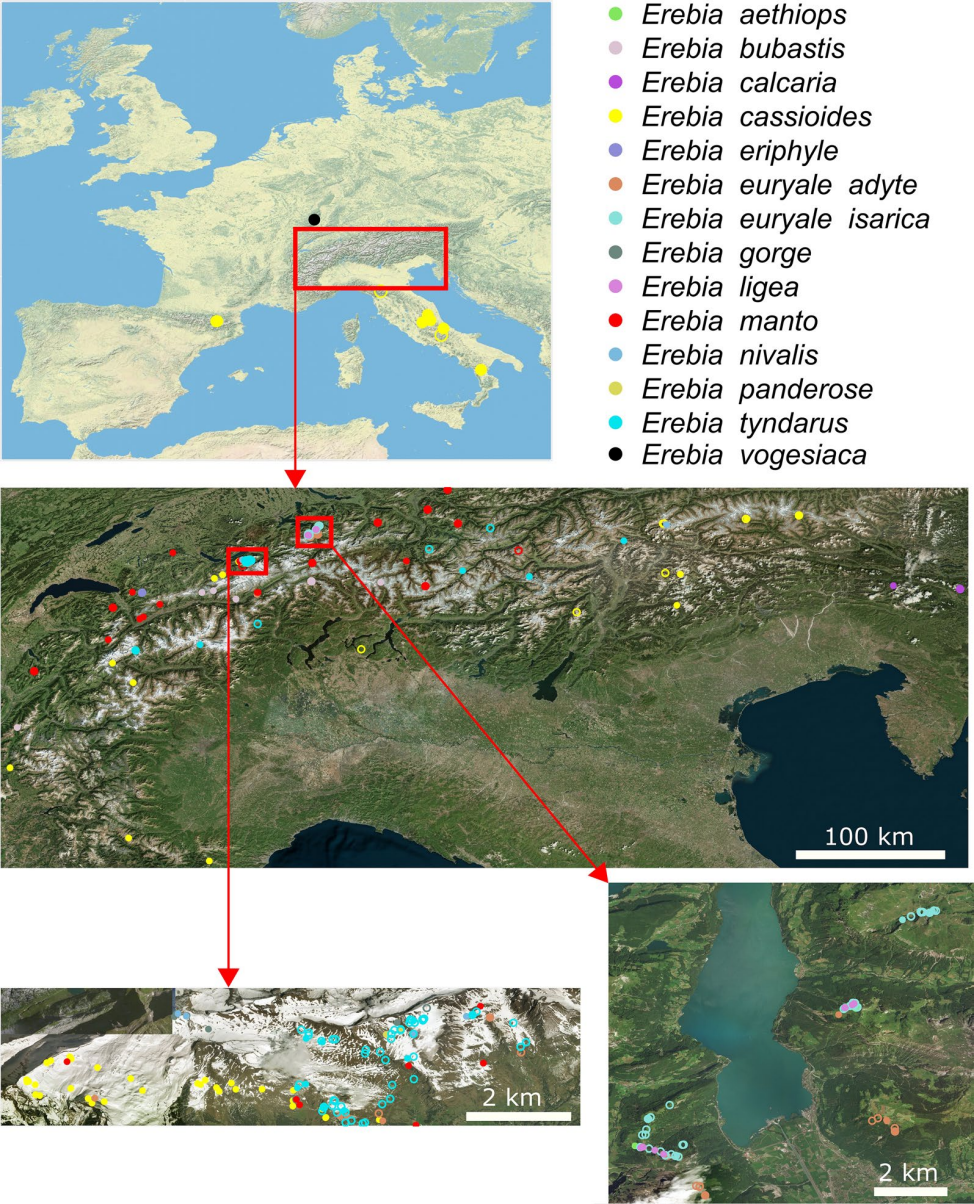
Map of Europe and the Alps depicting all sampled individuals. The two most densely sampled regions are further depicted in detail. Species are shown in different colors, where closed and open circles depict individuals with or without *Wolbachia* respectively (see also Additional file 2: Table S2). The map of Europe was obtained and modified from the U.S. National Parks Service—https://www.nps.gov. All other maps were obtained and modified from Bing Maps https://www.bing.com. All map data was retrieved via the OpenStreetMap package in R—https://github.com/ifellows/ROSM

We used genomic sequence data from restriction-site associated DNA (RAD) sequencing to estimate the prevalence of *Wolbachia* infections among different *Erebia* host species and to compare the phylogenetic relationship between *Wolbachia* and its hosts. Individuals from 13 recognized *Erebia* species or, in the case of *E. euryale,* distinct lineages that originate from different glacial refugia (*E. euryale ssp. adyte* and *E. euryale ssp. isarica*) were included. Most individuals were collected from the Alps with a main sampling focus on Switzerland (Fig. 2). Exceptions were *E. vogesiaca* that were sampled in the Vosges (France) and few *E. cassioides* that were collected in the Apennines (Italy) or Pyrenees (France, Spain) [28]. Because former studies suggested that populations of *E. cassioides* from geographically distinct mountain ridges likely originate from different glacial refugia [18, 23, 28], we treated them as separate lineages in the subsequent analyses.

**Fig. 3:**
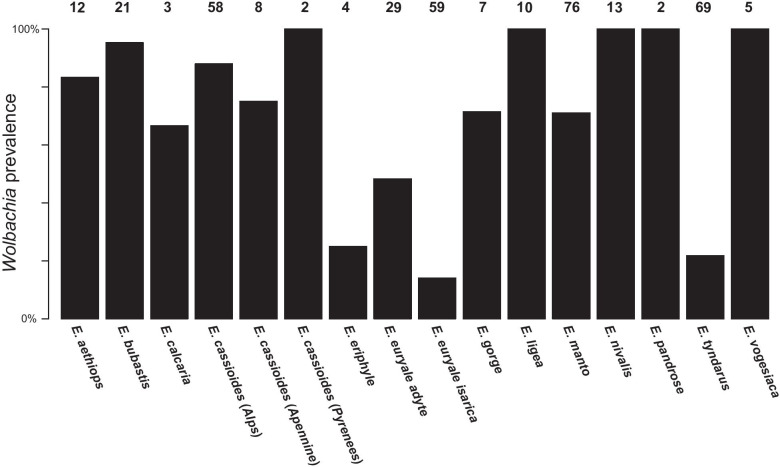
Prevalence (%) of *Wolbachia* among 376 *Erebia* host specimens. Numbers above bars indicate sample size

We detected *Wolbachia* in at least one individual of all our studied host species and lineages, but the prevalence varied among taxa (range 14%-100% of the individuals per species, mean 72.8%, SD 30.2%; Fig. 3; Additional file 2: Table S2). Overall, out of the 376 genotyped *Erebia*, we detected genomic evidence of an infection by *Wolbachia* in 218 (58.0%) individuals. The prevalence was particularly low in the subspecies *isarica* of *E. euryale* (14%, Fig. 2) as well as in *E. tyndarus*, confirming earlier observations [18]. For *E. cassioides*—the host species of our sequenced *Wolbachia* genome—the *Wolbachia* prevalence was high for specimens from the Alps (87.9%). Prevalence was also high throughout its range for individuals from the Apennines (75.0%) and Pyrenees (100%), but sample sizes were limited.

**Fig. 4:**
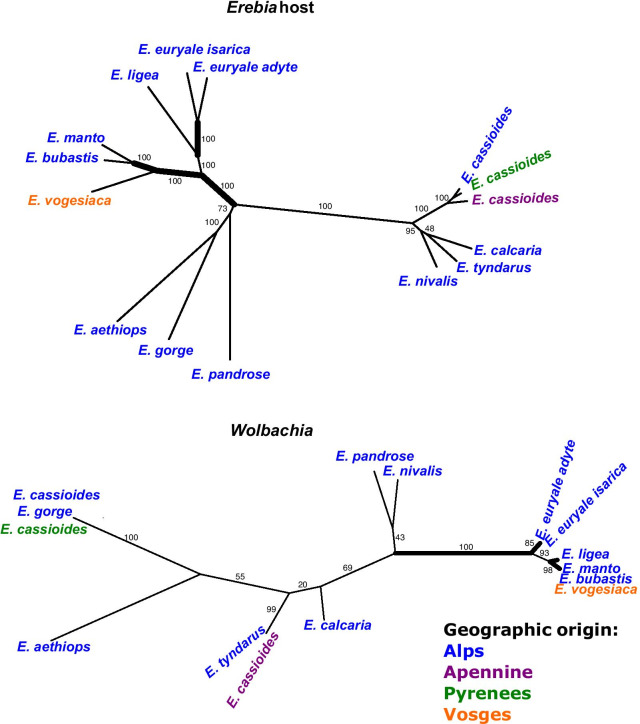
Unrooted phylogenetic relationship based on RAxML trees from polymorphic SNPs of *Erebia* and their associated *Wolbachia* strains. Numbers indicate bootstrap branch support and colors the geographic origin of the sampled specimens (see also Additional file 2: Table S2). Thick lines indicate shared splits as inferred by the function *comparePhylo* of the *ape* package in R

**Fig. 5:**
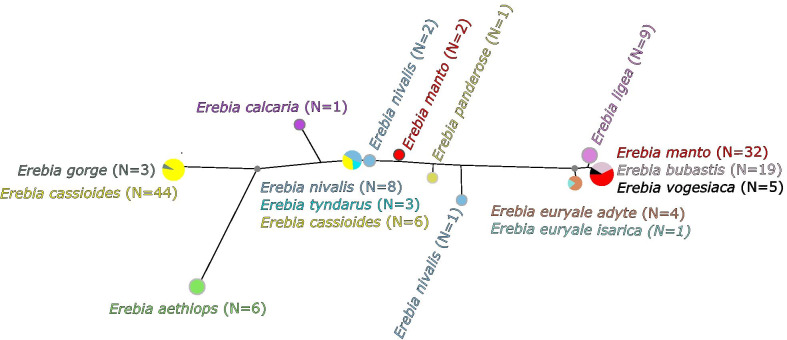
Unrooted phylogenetic relationship based on polymorphic SNPs for *Wolbachia* from 147 *Erebia* specimens (see Additional file 2: Table S2). Pie charts at each terminal node depict the frequency of *Erebia* host species carrying a given *Wolbachia* haplotype. Gray dots indicate nodes with > 95% bootstrap support

Following the mapping of all RAD sequence reads against the reference genomes and the subsequent SNP filtering, a total of 90 and 4,272 polymorphic nucleotide positions were respectively retained for *Wolbachia* and *Erebia*. Both resulting phylogenetic trees showed reasonable support (Fig. 4), where especially the phylogenetic relationships among *Erebia* hosts were well resolved. Although the topologies of hosts and *Wolbachia* strains differed at all but four nodes (two deep and two shallow nodes, i.e., 69.3% of the nodes were incongruent; Fig. 4), the *ParaFit* test [29] supports a certain level of coevolution by rejecting a random phylogenetic association between *Wolbachia* and *Erebia* (*p* = 0.016). Coevolution seems to principally occur between closely related hosts, e.g. between *E. euryale ssp. adyte* and *isarica* as well as between *E. bubastis, E. manto* and *E. vogesiaca*, that each shared the same *Wolbachia* strain (Fig. 4). The tree topologies further suggest additional scenarios including differentiation in *Wolbachia* strains due to different historical contingencies resulting from different glacial refugia. This seems to be the case for *E. cassioides*, where individuals from the Apennines harbored a *Wolbachia* strain that was distinct from the one found in the individuals from the Alps and Pyrenees but which also occurs in *E. tyndarus* and *E. nivalis* [18]. Another scenario are host shifts between the distantly related species, which we observed between *E. gorge* and *E. cassioides* and between *E. nivalis* and *E. pandrose*, which geographically overlap in the Alps and can share similar *Wolbachia* strains. By expanding our phylogenetic analysis to include all 147 *Wolbachia* genotypes that passed our filtering, we identified another case of host shift in *E. manto*, where two individuals are infected by a strain close to the one found in *E. tyndarus, E. nivalis* and some *E. cassioides* (Fig. 5; Additional file 2: Table S2).

## Discussion

While comparative studies between *Wolbachia* and their hosts suggest widespread horizontal transmission among arthropods, the factors that have shaped the current prevalence and diversity of *Wolbachia* often remain a conundrum [30]. Here, we first characterized the genome of *Wolbachia* infecting *Erebia cassioides* (Fig. 1; Additional file 1: Figure S1) and then assessed the occurrence and diversity of *Wolbachia* at an intermediate phylogenetic level, namely among several host species and glacial lineages across the genus *Erebia.* With a comparison among annotated *Wolbachia* genomes (Fig. 1) and all available Multi-Locus Sequence Typing (MLST) loci from arthropod hosts (Additional file 1: Figures S2–S6), we confirmed former studies, i.e. that *Wolbachia* strains often occur independently of the broader taxonomic relationship of their host species, consistent with prevalent horizontal transmissions events [30, 31]. Comparing whole genomes, our newly sequenced *Wolbachia* strain was close to the strain found in the moth *Operophtera brumata* (order Lepidoptera, Fig. 1), while the next closest strain is *wPip,* found in mosquitoes of the genus *Culex* (Additional file 2: Table S1). Addressing this question using MLST loci, we found *Wolbachia* from *E. cassioides* to be most closely related to *Wolbachia* strains found in other but very distantly related butterfly host species rather than in the few previously genotyped *Erebia* specimens (Additional file 1: Figures S3–S5). Interestingly, for three MLST loci the closest lineage we found was in spider mites (Acari: Trombidiformes; Additional file 1: Figures S2–S6). This result might implicate that mites cause *Wolbachia* host switches in Lepidoptera, as mites are common parasites for Lepidoptera [32] and have been suggested to act as potential vectors for *Wolbachia* in other arthropod lineages [33]*.* However, currently only non-parasitic mites have been genotyped for *Wolbachia*. Both analyses suggest that our newly sequenced *Wolbachia* genome belongs to the supergroup B (Fig. 1; Additional file 1: Figures S2–S6), which is widespread among Lepidoptera and has previously been identified in three *Erebia* species from Siberia [10]. *Wolbachia* of supergroup B can affect their hosts in various ways [31] but their potential impact in *Erebia* need further investigation.

*Wolbachia* has been shown to be abundant among butterfly hosts [12] but only few comparative studies on closely related species within a genus and/or within a close geographic range exist (e.g. [3, 10, 12]). Turnovers in *Wolbachia* infections from uninfected to infected and vice versa are common among host taxa, where infected species may lose *Wolbachia* slightly more often than uninfected species acquire it, which has been suggested to represent a state of epidemiological equilibrium [3]. Intraspecific fluctuations in *Wolbachia* prevalence may also occur, e.g. through a loss during range expansions due to stochastic processes such as drift or founder effects [34, 35], the loss of cytoplasmic incompatibility in *Wolbachia* [36] or due to environmental factors [35, 37]. Similarly, novel strains may also be acquired in the newly colonized ranges [34, 35]. Most studies on *Wolbachia* diversity use MLST loci for which an extensive reference database for a set of standard genes exist [38]. For our broader survey of *Wolbachia* in *Erebia*, we relied on a dataset generated by restriction-site associated DNA (RAD) sequencing. RAD sequencing is commonly used for population genetic studies of host species [39]. While our approach allows us to also confidently identify the infection status of a host specimen, the resulting data is not compatible with traditional MLST genotyping, as the RAD loci did not overlap with the MLST loci.

With two exceptions, we found the prevalence of *Wolbachia* to be relatively high (> 75%) among our studied *Erebia* taxa and lineages (Fig. 3). The first exception is *E. tyndarus* for which *Wolbachia* was primarily found in individuals from its distribution in the eastern Alps [18], closer to its putative glacial refugium [23]. These individuals account for most of the observed *Wolbachia* infections in our study (Additional file 2: Table S2), indicating that *E. tyndarus* may have lost *Wolbachia* during postglacial range expansion. The second exception is *E. euryale ssp. isarica,* for which similar processes may be at play, given that the two included *E. euryale* lineages originate from distinct glacial refugia [40] and were mainly sampled at the zones of secondary contact between *adyte* and *isarica* representing their range edges. Further sampling along a transect from the putative glacial refugia to the zones of secondary contact is however needed to understand how *Wolbachia* could have been lost during range expansion in these two cases. Importantly, this reduction or in the case of *E. tyndarus* the almost complete loss of *Wolbachia* at the expansion fronts may also suggest that the potential for horizontal transmission could be limited despite co-occurring *Erebia* species [19]*.* This could for instance reflect differences in micro-habitat use [24] or differences in immunity [41] but requires further formal testing.

Coevolution has been commonly invoked to shape the association of *Wolbachia* and its hosts [42, 43] and is often inferred by cophylogenetic approaches [29, 44]. For our studied *Erebia* host species, we observed phylogenetic relationships that are congruent with former studies, which included more taxa but were based on only few sequenced genes [20, 25]. In addition to the described taxa, *E. cassioides* from distinct mountain ridges were significantly separated in our phylogeny, which is consistent with formerly described glacial lineages [28]*.* Comparing the phylogenetic relationships of *Erebia* with that of their *Wolbachia* strains, we overall found significant statistical support for some level of coevolution. The latter comes from the three closely related species *E. manto*, *E. bubastis* and *E. vogesiaca,* where the phylogenetic relationships of the host species are congruent with those observed among their parasites (Fig. 4). This is striking as the current geographic distribution of the host taxa is rather broad, mostly allopatric, and could suggest a recent diversification from a common ancestor for both the hosts and *Wolbachia* in this group [19, 45]. Coevolution may similarly have occurred in *E. euryale,* but most of our sampled individuals were collected at a contact site between *adyte* and *isarica,* where intermediate phenotypes exist and thus recent gene flow between the two lineages occurs [46].

In addition to coevolution between closely related host taxa, we found evidence for host shifts, i.e., where similar *Wolbachia* strains occur in phylogenetically distinct host species or distinct *Wolbachia* strains within the same host species. This is indicated in at least three cases. The first is for the *Wolbachia* strain associated with *E. gorge* that is most closely related with a strain found in the otherwise very distantly related host *E. cassioides.* The second occurs between *E. nivalis* and *E. pandrose* (Fig. 4). Thirdly, we found two individuals of *E. manto* that were infected by a *Wolbachia* strain similar to the one found in members of the *tyndarus* group (Fig. 5), while other *E. manto* that were collected at the same locations were infected by the strain that commonly infects *E. manto*, *E. bubastis* and *E. vogesiaca* (Additional file 2: Table S2). Intraspecific variation in *Wolbachia* strains has also been reported for other species [34, 47] including *Erebia* [10], and may for example result in a dynamic pattern of *Wolbachia* infections due to different competing strains [47]. How these may manifest in *E. manto* requires further investigation by genotyping more host individuals.

Lastly, historical contingencies associated with different glacial refugia of the host species seem to also account for the diversity in *Wolbachia*. In the present dataset, this is exemplified by the two distinct *Wolbachia* strains in *E. cassioides*—one from Italy that is closely related to the strain of *E. tyndarus* and another found in the Western Alps and the Pyrenees (Fig. 3). This is consistent with two distinct glacial refugia of the *Erebia* hosts [18, 23, 28] and indicates that past demographic history further shapes the current diversity and occurrence of *Wolbachia.*

## Conclusions

Taken together, our study uncovered part of the hidden diversity of *Wolbachia* in *Erebia* even though we only had data for a fraction of the potential *Erebia* host taxa and biogeographic ranges [20]. Therefore, future broad scale studies in this genus are likely to advance our knowledge on the dynamic associations of *Wolbachia* and its butterfly host. The current associations between *Wolbachia* and their hosts seem to be a result of different processes, including phylogenetic coevolution and geographically restricted host shifts among more distant lineages. We also show that differentiation in distinct glacial refugia has likely shaped some of the current associations between *Wolbachia* and *Erebia*. Divergent *Wolbachia* infections can have profound evolutionary implications for their hosts, for example by acting as a barrier to gene flow [16], as has been suggested for *E. tyndarus* and *E. cassioides* [18]*.* However, the strain that we identified belongs to the widespread *Wolbachia* supergroup B, which can affect its hosts in various ways and cause reproductive isolation [48]. Given that the evolutionary implications of *Wolbachia* are best studied in groups of closely related taxa [47, 49], *Erebia* provides an excellent system to assess these. From a technical perspective, our study shows that genomic markers commonly used to study the population genetic structure of host species can also be used to study the prevalence of endoparasitic infections. Indeed, studies performing whole genome sequencing in arthropods also sequence the genome *Wolbachia*, allowing obtaining a genome-scale perspective on *Wolbachia* evolution [30].

## Methods

### Wolbachia genome sequencing & analyses

We collected a female specimen of *Erebia cassioides* (Reiner & Hohenwarth 1792) in September 2017 in Grindelwald, Switzerland (46.65995°N, 8.00443°E), which we flash froze. DNA was extracted from all available tissue, except the abdomen using the Qiagen MagAttract HMW DNA Kit (Qiagen, Zug, Switzerland) following the manufacturer’s protocol. We then sequenced its DNA on a PacBio Sequel platform (Pacific Biosystems, Menlo Park, CA, USA) using four PacBio SMRT cells. Library preparation and sequencing followed the manufacturer’s protocol and was done by the Genomics Facility Basel, (ETH-Zurich, Basel, Switzerland).

All obtained reads were used for assembly with the PacBio SMRT-Link genome assembly pipeline version 7.0.1.66975 [50]. We subsequently polished the assembly with four runs of *arrow* (part of the SMRT-Link pipeline), removing haplotigs with the setting *purge_haplotigs* [51]*,* followed by two runs of *pilon* 1.23 [52]. Next, we identified *Wolbachia* related contigs by comparing each contig against the NCBI nucleotide collection on February 23^rd^ 2020 using Blast + 2.9.0 [53]. To annotate the retained *Wolbachia* contigs, we used the Rapid Annotation using Subsystem Technology (Rast) web service version 2.0 [54]. We estimated the completeness of our *Wolbachia* genome with the Busco pipeline 3.0.2 [55], which was performed against the *proteobacteria_odb9* database containing 221 BUSCO groups of highly conserved single copy orthologs obtained from 1520 proteobacteria species.

In a first step, we established the phylogenetic relationship of our sequenced *Wolbachia* genome. For this we downloaded 34 annotated *Wolbachia* genomes of other insect hosts as well as one nematode host (*Pratylenchus penetrans*) that were available in March 2020  from the NCBI Genome Database (www.ncbi.nlm.nih.gov/genome; Additional file 2: Table S1). The included genomes covered five of the 16 known *Wolbachia* supergroups (i.e. groups A, B, E, F and L; [56]). We identified all single copy orthologs across all *Wolbachia* genomes with OrthoFinder [57]. DNA coding sequences of each ortholog were then aligned with Mafft v.7 [58] using default settings. After manual inspection of a representative set of alignments, we generated maximum-likelihood gene trees using RAxML 8.2.11 [59] assuming a GTRCAT substitution model, followed by a thorough maximum likelihood search using GTRGAMMA. Node support was computed from 100 bootstrap replicates. To generate a *Wolbachia* lineage tree from the gene trees, we used the coalescent-based species tree software Astral 5.7.3 [60], with 100 bootstrap replicates to compute the local posterior probability node support.

In a second step we identified the supergroup identity of our sequenced *Wolbachia* genome by extracting the sequences of five common MLST loci: *coxA*, *fbpA*, *ftsZ*, *gatB* and *hcpA*. For the same loci, we downloaded on the 17th of April 2021 all (N = 767) accessions from PubMLST (www.pubmlst.org) that were sequenced from arthropod hosts for which the host species was identified to the species level and all five loci were sequenced. For each MLST locus, we identified unique alleles (range N: 372–498; Additional file 1: Figures S2–S6) and used these to generate maximum-likelihood gene trees with RAxML. From each gene tree, we determined the set of alleles that was most closely related to the newly sequenced allele.

### Wolbachia abundance & coevolution

We genotyped a total of 237 individuals using single-end restriction-site associated DNA (RAD) sequencing with the restriction enzyme *Sbf*I (Additional file 2: Table S2). Data for another 139 individuals was moreover available from two published datasets ([18, 28], Additional file 2: Table S2). For all newly genotyped individuals, we extracted DNA from thorax tissue using the Qiagen DNeasy Blood & Tissue Kit following the manufacturer’s protocol. Library preparation and sequencing was outsourced to Floragenex (Portland, OR, USA).

To estimate the prevalence of *Wolbachia* among *Erebia,* we first mapped the reads of each individual against our newly generated *Wolbachia* genome assembly with Bwa Mem 0.7.17 [61]. To identify individuals that had mapping reads or not, we genotyped all specimens with BCFtools 1.10.2 [62].

To compare the phylogenetic relationship of *Wolbachia* and their *Erebia* hosts, we selected for each taxonomic group the specimen that had the lowest amount of missing data. *E. eriphyle* had to be excluded from the phylogenetic analyses given the high fraction of missing data. We filtered the genotypes with VCFtools 0.1.16 [63] to include only bi-allelic polymorphic sites with a minimal depth of six and a minimal genotype quality of 28, allowing up to 50% of missing data per site. For the same *Erebia* host specimens, we aligned all reads against an *Erebia* draft assembly and performed SNP calling as for *Wolbachia*. Subsequent SNP filtering was the same, except that we employed a minor allele-frequency filtering of 0.03 and allowed only 25% missing data. For each filtered SNP dataset, we constructed a phylogenetic hypothesis using maximum likelihood, because the SNP data did not allow reconstructing individual gene trees. We used RAxML 8.2.11, implementing a GTRGAMMA substitution model and corrected for ascertainment bias as we only used polymorphic SNP positions with the Asc_gtrgamma function. Node support was assessed using 1000 bootstrap replicates followed by a thorough maximum likelihood search. Using the *ape* package [64] in R 3.5.1 [65], we first compared the resulting phylogenetic trees with the function *comparePhylo.* We then tested for coevolution between *Wolbachia* and their *Erebia* hosts using the *ape* function *ParaFit*, which implements a matrix permutation test of coevolution [29]. The significance of this signal was assessed with 1000 permutations. To further assess if multiple *Wolbachia* strains may occur within a species, we similarly constructed a phylogenetic hypothesis comprising data from 147 host individuals that passed the abovementioned filtering (Additional file 2: Table S2) using the same RAxML parameters.

## Supplementary Information

**Additional file 1.** Additional figures S1–S6.

**Additional file 2.** **Table S1.** Summary table of all annotated Wolbachia genomes used in this study including the newly sequenced strain from *E. cassioides*. For each genome the taxonomic. **Table S2.** Summary data for all genotyped individuals.

## Data Availability

The assembly of the newly sequenced *Wolbachia* strain as well as all newly sequenced RAD data are available from NCBI (BioProjects PRJNA691873,  PRJNA729987, PRJNA730041). The single copy orthologs and the MLST alignments are available from Zenodo (10.5281/zenodo.4766332).

## Abbreviations

BUSCO: Benchmarking Universal Single-Copy Orthologs
HMW: High Molecular Weight
MLST: Multi-Locus Sequence Typing
NCBI: National Center for Biotechnology Information
RAD: Restriction site Associated DNA
RAST: Rapid Annotation using Subsystem Technology
SD: Standard Deviation
SNP: Single Nucleotide Polymorphism

## References

[1] Weinert LA, Araujo-Jnr EV, Ahmed MZ, Welch JJ (2015). The incidence of bacterial endosymbionts in terrestrial arthropods. P R Soc B.

[2] Werren JH, Howard DJ, Berlocher SH (1998). *Wolbachia* and speciation. Endless forms: species and speciation.

[3] Bailly-Bechet M, Martins-Simões P, Szöllősi GJ, Mialdea G, Sagot M-F, Charlat S (2017). How long does *Wolbachia* remain on board?. Mol Biol Evol.

[4] Jiggins FM, Hurst GDD, Dolman CE, Majerus MEN (2000). High-prevalence male-killing *Wolbachia* in the butterfly *Acraea encedana*. J Evol Biol.

[5] Fukui T, Kawamoto M, Shoji K, Kiuchi T, Sugano S, Shimada T, Suzuki Y, Katsuma S (2015). The endosymbiotic bacterium *Wolbachia* selectively kills male hosts by targeting the masculinizing gene. PLoS Pathog..

[6] Bordenstein SR, O'Hara FP, Werren JH (2001). *Wolbachia*-induced incompatibility precedes other hybrid incompatibilities in *Nasonia*. Nature.

[7] Bossan B, Koehncke A, Hammerstein P (2011). A new model and method for understanding *Wolbachia*-induced cytoplasmic incompatibility. PLoS ONE.

[8] Sumi T, Miura K, Miyatake T (2017). *Wolbachia* density changes seasonally amongst populations of the pale grass blue butterfly, *Zizeeria maha* (Lepidoptera: Lycaenidae). PLoS ONE.

[9] Tseng S-P, Wetterer JK, Suarez AV, Lee C-Y, Yoshimura T, Shoemaker D, Yang C-C (2019). Genetic diversity and *Wolbachia* infection patterns in a globally distributed invasive ant. Front Genet.

[10] Ilinsky Y, Kosterin OE (2017). Molecular diversity of *Wolbachia* in Lepidoptera: Prevalent allelic content and high recombination of MLST genes. Mol Phylogenet Evol.

[11] Stork NE (2018). How many species of insects and other terrestrial arthropods are there on Earth?. Annu Rev Entomol.

[12] Ahmed MZ, Araujo-Jnr EV, Welch JJ, Kawahara AY (2015). *Wolbachia* in butterflies and moths: geographic structure in infection frequency. Front Zool..

[13] Kageyama D, Ohno M, Sasaki T, Yoshido A, Konagaya T, Jouraku A (2017). Feminizing *Wolbachia* endosymbiont disrupts maternal sex chromosome inheritance in a butterfly species. Evol Lett.

[14] Dyson EA, Hurst GDD (2004). Persistence of an extreme sex-ratio bias in a natural population. P Natl Acad Sci USA.

[15] Sasaki T, Kubo T, Ishikawa H (2002). Interspecific transfer of *Wolbachia* between two Lepidopteran insects expressing cytoplasmic incompatibility: a *Wolbachia* variant naturally infecting *Cadra cautella* causes male killing in *Ephestia kuehniella*. Genetics.

[16] Telschow A, Hilgenboecker K, Hammerstein P, Werren JH (2014). Dobzhansky-Muller and *Wolbachia*-induced incompatibilities in a diploid genetic system. PLoS ONE.

[17] Lis A, Maryańska-Nadachowska A, Kajtoch Ł (2015). Relations of *Wolbachia* infection with phylogeography of *Philaenus spumarius* (Hemiptera: Aphrophoridae) populations within and beyond the Carpathian contact zone. Microb Ecol.

[18] Lucek K, Butlin RK, Patsiou T (2020). Secondary contact zones of closely-related *Erebia* butterflies overlap with narrow phenotypic and parasitic clines. J Evol Biol.

[19] Sonderegger P (2005). Die Erebien der Schweiz.

[20] Peña C, Witthauer H, Kleckova I, Fric Z, Wahlberg N (2015). Adaptive radiations in butterflies: evolutionary history of the genus *Erebia* (Nymphalidae: Satyrinae). Biol J Linn Soc.

[21] Schmitt T, Hewitt GM, Müller P (2006). Disjunct distributions during glacial and interglacial periods in mountain butterflies: *Erebia epiphron* as an example. J Evol Biol.

[22] Schmitt T, Haubrich K (2008). The genetic structure of the mountain forest butterfly *Erebia euryale* unravels the late Pleistocene and postglacial history of the mountain coniferous forest biome in Europe. Mol Ecol.

[23] Schmitt T, Louy D, Zimmermann E, Habel JC (2016). Species radiation in the Alps: multiple range shifts caused diversification in ringlet butterflies in the European high mountains. Org Div Evol.

[24] Kleckova I, Konvicka M, Klecka J (2014). Thermoregulation and microhabitat use in mountain butterflies of the genus *Erebia*: importance of fine-scale habitat heterogeneity. J Therm Biol.

[25] de Vos JM, Augustijnen H, Bätscher L, Lucek K (2020). Speciation through chromosomal fusion and fission in Lepidoptera. Phil Trans R Soc B.

[26] Sinha A, Li Z, Sun L, Carlow CKS (2019). Complete genome sequence of the *Wolbachia wAlbB* endosymbiont of *Aedes albopictus*. Genome Biol Evol.

[27] Derks MFL, Smit S, Salis L, Schijlen E, Bossers A, Mateman C, Pijl AS, de Ridder D, Groenen MAM, Visser ME, Megens HJ (2015). The genome of winter moth (*Operophtera brumata*) provides a genomic perspective on sexual dimorphism and phenology. Genome Biol Evol.

[28] Gratton P, Trucchi E, Trasatti A, Riccarducci G, Marta S, Allegrucci G, Cesaroni D, Sbordoni V (2016). Testing classical species properties with contemporary data: How “bad species” in the brassy ringlets (*Erebia tyndarus* complex, Lepidoptera) turned good. Syst Biol.

[29] Legendre P, Desdevises Y, Bazin E (2002). A statistical test for host-parasite coevolution. Syst Biol.

[30] Scholz M, Albanese D, Tuohy K, Donati C, Segata N, Rota-Stabelli O (2020). Large scale genome reconstructions illuminate *Wolbachia* evolution. Nat Commun.

[31] Bing X-L, Zhao D-S, Sun J-T, Zhang K-J, Hong X-Y (2020). Genomic analysis of *Wolbachia* from *Laodelphax striatellus* (Delphacidae, Hemiptera) reveals insights into its “Jekyll and Hyde” mode of infection pattern. Genome Biol Evol.

[32] Conradt L, Corbet SA, Roper TJ, Bodsworth EJ (2002). Parasitism by the mite *Trombidium breei* on four UK butterfly species. Ecol Entomol.

[33] Sanaei E, Charlat S, Engelstädter J (2021). *Wolbachia* host shifts: routes, mechanisms, constraints and evolutionary consequences. Biol Rev.

[34] Reuter M, Pedersen JS, Keller L (2004). Loss of *Wolbachia* infection during colonisation in the invasive Argentine ant *Linepithema humile*. P R Soc B.

[35] Nguyen DT, Spooner-Hart RN, Riegler M (2015). Loss of *Wolbachia* but not *Cardinium* in the invasive range of the Australian thrips species *Pezothrips kellyanus*. Biol Invasions.

[36] Meany MK, Conner WR, Richter SV, Bailey JA, Turelli M, Cooper BS (2019). Loss of cytoplasmic incompatibility and minimal fecundity effects explain relatively low *Wolbachia* frequencies in *Drosophila mauritiana*. Evolution.

[37] Rey O, Estoup A, Facon B, Loiseau A, Aebi A, Duron O (2013). Distribution of endosymbiotic reproductive manipulators reflects invasion process and not reproductive system polymorphism in the little fire ant *Wasmannia auropunctata*. PLoS ONE.

[38] Baldo L, Hotopp J, Jolley K, Bordenstein SR, Biber S, Raychoudhury R (2006). Multilocus sequence typing system for the endosymbiont *Wolbachia pipientis*. Appl Env Microbiol..

[39] Catchen JM, Hohenlohe PA, Bernatchez L, Funk WC, Andrews KR, Allendorf FW (2017). Unbroken: RADseq remains a powerful tool for understanding the genetics of adaptation in natural populations. Mol Ecol Resour.

[40] Cupedo F (2014). Reproductive isolation and intraspecific structure in Alpine populations of *Erebia euryale* (Esper, 1805) (Lepidoptera, Nymphalidae, Satyrinae). Nota Lepi.

[41] Pigeault R, Braquart-Varnier C, Marcadé I, Mappa G, Mottin E, Sicard M (2014). Modulation of host immunity and reproduction by horizontally acquired *Wolbachia*. J Insect Physiol.

[42] Lefoulon E, Bain O, Makepeace BL, d’Haese C, Uni S, Martin C, Gavotte L (2016). Breakdown of coevolution between symbiotic bacteria *Wolbachia* and their filarial hosts. PeerJ.

[43] Kajtoch Ł, Kolasa M, Kubisz D, Gutowski JM, Ścibior R, Mazur MA, Holecova H (2019). Using host species traits to understand the *Wolbachia* infection distribution across terrestrial beetles. Sci Rep.

[44] de Vienne DM, Refrégier G, López-Villavicencio M, Tellier A, Hood ME, Giraud T (2013). Cospeciation vs host-shift speciation: methods for testing, evidence from natural associations and relation to coevolution. New Phytol.

[45] Schmitt T, Habel JC, Rödder D, Louy D (2014). Effects of recent and past climatic shifts on the genetic structure of the high mountain yellow-spotted ringlet butterfly *Erebia manto (*Lepidoptera, Satyrinae): a conservation problem. Glob Chang Biol.

[46] Rezbanyai-Reser L (1991). Die drei Zentralschweizer Kontaktstellen der *Erebia euryale* Unterarten *isarica* HEYNE und *adyte* HBN. Entomo Ber Luzern.

[47] Raychoudhury R, Baldo L, Oliveira DCSG, Werren JH (2009). Modes of acquisition of *Wolbachia*: horizontal transfer, hybrid introgression, and codivergence in the *Nasonia* species complex. Evolution.

[48] Ellegaard KM, Klasson L, Näslund K, Bourtzis K, Andersson SGE (2013). Comparative genomics of *Wolbachia* and the bacterial species concept. PLoS Genet..

[49] Richardson KM, Griffin PC, Lee SF, Ross PA, Endersby-Harshman NM, Schiffer M, Hoffmann AA (2019). A *Wolbachia* infection from *Drosophila* that causes cytoplasmic incompatibility despite low prevalence and densities in males. Heredity.

[50] Chin C-S, Alexander DH, Marks P, Klammer AA, Drake J, Heiner C, Clum A, Copeland A, Huddleston J, Eichler EE, Turner SW, Korlach J (2013). Nonhybrid, finished microbial genome assemblies from long-read SMRT sequencing data. Nat Methods.

[51] Roach MJ, Schmidt SA, Borneman AR (2018). Purge Haplotigs: allelic contig reassignment for third-gen diploid genome assemblies. BMC Bioinformatics.

[52] Walker BJ, Abeel T, Shea T, Priest M, Abouelliel A, Sakthikumar S, Cuomo CA, Zheng Q, Wortmann J, Young SK (2014). Pilon: an integrated tool for comprehensive microbial variant detection and genome assembly improvement. PLoS ONE.

[53] Camacho C, Coulouris G, Avagyan V, Ma N, Papadopoulos J, Bealer K, Madden TL (2009). BLAST+: architecture and applications. BMC Bioinformatics.

[54] Aziz RK, Bartels D, Best AA, DeJongh M, Disz T, Edwards RA (2008). The RAST server: rapid annotations using subsystems technology. BMC Genomics.

[55] Simão FA, Waterhouse RM, Ioannidis P, Kriventseva EV, Zdobnov EM (2015). BUSCO: assessing genome assembly and annotation completeness with single-copy orthologs. Bioinformatics.

[56] Driscoll TP, Verhoeve VI, Brockway C, Shrewsberry DL, Plumer M, Sevdalis SE (2020). Evolution of *Wolbachia* mutualism and reproductive parasitism: insight from two novel strains that co-infect cat fleas. PeerJ.

[57] Emms DM, Kelly S (2019). OrthoFinder: phylogenetic orthology inference for comparative genomics. Genome Biol.

[58] Katoh K, Standley DM (2013). MAFFT multiple sequence alignment software version 7: improvements in performance and usability. Mol Biol Evol.

[59] Stamatakis A (2014). RAxML version 8: a tool for phylogenetic analysis and post-analysis of large phylogenies. Bioinformatics.

[60] Zhang C, Rabiee M, Sayyari E, Mirarab S (2018). ASTRAL-III: polynomial time species tree reconstruction from partially resolved gene trees. BMC Bioinformatics.

[61] Li H. Aligning sequence reads, clone sequences and assembly contigs with BWA-MEM. arXiv.org. 2013. http://arxiv.org/abs/1303.3997. Accessed 27 June 2016.

[62] Li H (2011). A statistical framework for SNP calling, mutation discovery, association mapping and population genetical parameter estimation from sequencing data. Bioinformatics.

[63] Danecek P, Auton A, Abecasis G, Albers CA, Banks E, DePristo MA (2011). The variant call format and VCFtools. Bioinformatics.

[64] Paradis E, Schliep K (2019). ape 5.0: an environment for modern phylogenetics and evolutionary analyses in R. Bioinformatics.

[65] The R Core Team. R 3.5.1: A language and environment for statistical computing. Vienna: R Foundation for Statistical Computing. https://www.R-project.org/. Acessed 1 August 2018.

