# Supplementary material for: Prevalence and relationship of endosymbiotic Wolbachia in the butterfly genus Erebia
Source: BMC Ecol Evol. 2021 May 21;21:95. doi: 10.1186/s12862-021-01822-9 (PMC8140509; doi:10.1186/s12862-021-01822-9)
Supplement: Supplementary file 1 — Additional file 1. Additional figures S1–S6. [file 12862_2021_1822_MOESM1_ESM.docx]

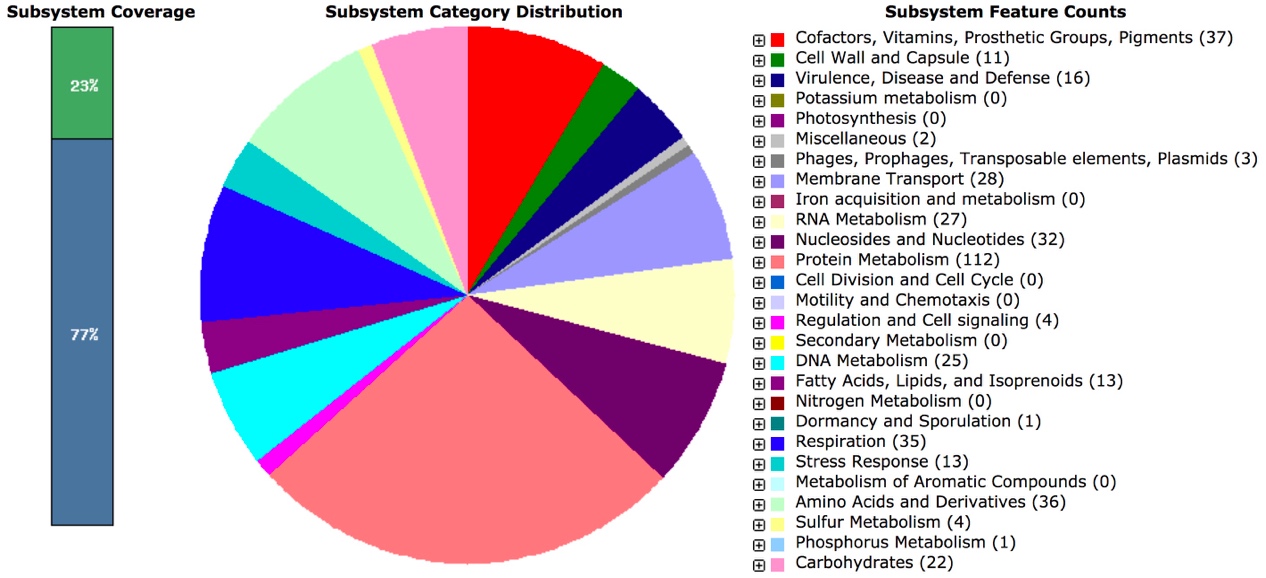


Figure S1: Summary of annotation for the *Wolbachia* strain of *Erebia* *cassioides* based on RAST subsystem.

Figure S2: RAxML gene tree arbitrarily rooted at the stem lineage of supergroup F for the MLST locus *coxA* based on 424 unique alleles. The first letter indicates the *Wolbachia* supergroup followed by the taxonomic order, family and species name, its geographic location and MLST id as taken from the PubMLST database. Finally, the unique allele id of this analysis is indicated. *Erebia cassioides* is highlighted in red.

Figure S3: RAxML gene tree arbitrarily rooted at the stem lineage of supergroup F for the MLST locus *fbpA* based on 410 unique alleles. The first letter indicates the *Wolbachia* supergroup followed by the taxonomic order, family and species name, its geographic location and MLST id as taken from the PubMLST database. Finally, the unique allele id of this analysis is indicated. *Erebia cassioides* is highlighted in red.

Figure S4: RAxML gene tree arbitrarily rooted at the stem lineage of supergroup F for the MLST locus *ftsZ* based on 372 unique alleles. The first letter indicates the *Wolbachia* supergroup followed by the taxonomic order, family and species name, its geographic location and MLST id as taken from the PubMLST database. Finally, the unique allele id of this analysis is indicated. *Erebia cassioides* is highlighted in red.

Figure S5: RAxML gene tree arbitrarily rooted at the stem lineage of supergroup F for the MLST locus *gatB* based on 442 unique alleles. The first letter indicates the *Wolbachia* supergroup followed by the taxonomic order, family and species name, its geographic location and MLST id as taken from the PubMLST database. Finally, the unique allele id of this analysis is indicated. *Erebia cassioides* is highlighted in red.

Figure S6: RAxML gene tree arbitrarily rooted at the stem lineage of supergroup F for the MLST locus *hcpA* based on 498 unique alleles. The first letter indicates the *Wolbachia* supergroup followed by the taxonomic order, family and species name, its geographic location and MLST id as taken from the PubMLST database. Finally, the unique allele id of this analysis is indicated. *Erebia cassioides* is highlighted in red.
